# How Conventional Visual Representations of Time-Frequency Analyses Bias Our Perception of EEG/MEG Signals and What to Do About It

**DOI:** 10.3389/fnhum.2019.00212

**Published:** 2019-06-25

**Authors:** Adrian P. Burgess

**Affiliations:** Department of Psychology, Life and Health Sciences, Aston University, Birmingham, United Kingdom

**Keywords:** time-frequency (TF) analysis, EEG & MEG, wavelet transform, bias (systemic error), visual representacion

Time-frequency decompositions of the EEG/MEG have become such a familiar part of the cognitive neuroscience landscape over the past two decades that their appearance no longer seems remarkable. But to those of us who labored in the days when the signal analysis toolbox contained Fourier analysis, event-related potentials and not much else, the arrival of time-frequency decompositions was little short of revolutionary. With their introduction, complex information about both the timing *and* frequency of changes in the EEG/MEG could be presented in the visually attractive format of time-frequency plots (TFPs). Like maps, with time on the abscissa, frequency on the ordinate and a color or gray scale to indicate the amplitude or power at each time-frequency location, TFPs provide a convenient and efficient way to represent a large amount of detailed information in an easily digestible format and, for that, they are to be commended. Yet, despite all these benefits, it is my contention that TFPs, in the format most commonly seen in journal articles and at conferences, systematically distort and bias our perception of the EEG/MEG signals that they are supposed to help us understand.

Specifically, my contention is that TFPs are biased by the use of linear frequency scales. Linear frequency scales distort our perception of the EEG/MEG signal by placing far too much emphasis on the high frequency components of the signal, where there is very little energy, and far too little emphasis on the lower frequencies where the biggest changes are seen. This disproportionate focus on high frequencies confers a degree of significance to the gamma band that is not justified by the evidence.

In the waking state, EEG/MEG spectral power approximately follows a $\frac{1}{f}$ distribution (Linkenkaer-Hansen et al., 2001), where *f* is the frequency, which means that power is greatest at low frequencies and diminishes rapidly as frequency increases. The consequence of this is that less than a quarter of EEG/MEG power from 1 to 80 Hz is in gamma (30–80 Hz) even though this spans close to two-thirds of the frequency range. In contrast, nearly half the power in the signal is in delta (1 to 4 Hz) and theta (4 to 8 Hz) which together cover <10% of the total frequency range. In short, the use of linear frequency scales is biased in that it massively over-represents the higher frequency ranges at the expense of the lower ones.

Signal power, of course, is not the only consideration and this disproportionate emphasis on higher frequencies could be justified if the most significant EEG/MEG frequency bands were particularly densely distributed in the higher frequency range, but they are not. Despite the widespread lack of consensus about anything to do with EEG/MEG frequency bands (even the number of bands that exist is disputed), the rule that bandwidth increases with frequency is near universally acknowledged, at least implicitly (Penttonen and Buzsáki, 2003; Roopun et al., 2008; Pletzer et al., 2010; Klimesch, 2013; Newson and Thiagarajan, 2018). If we consider the five classical frequency bands, delta is typically defined as 3 Hz wide (1–4 Hz), theta 4 Hz (4–8 Hz), alpha 5 Hz (8–13), beta 17 Hz (13–30 Hz), and gamma is variously defined as anything from 15 Hz (30–45 Hz) to 70 Hz (30–100 Hz) wide. Of course, definitions of the classical frequency bands vary widely (Newson and Thiagarajan, 2018) and each of are sometimes sub-divided into two or more sub-bands, but the general rule remains: the higher the frequency, the broader the band. The clear consequence of this is that the frequency bands, however many of them there are, are clustered in the lower part of the frequency range and this means that linear frequency scales over-represent the very parts of the time-frequency plane that are most sparsely populated by distinct frequency bands. This relative lack of variation at higher frequencies often appears on TFPs as a characteristic series of narrow vertical stripes in the gamma range that represent broad-spectrum, low amplitude oscillations

In some ways, TFPs with linear frequency scales are like Mercator projection maps. The Mercator projection is a means of representing the geography of the globe in two dimensions and produces the standard world map that we are all familiar with. Unfortunately, the Mercator projection is misleading in important ways for whilst it preserves angle, thereby reproducing the shape of the continents accurately, it massively distorts area. For example, Greenland and Africa appear to be of similar size when in reality Africa is 14 times larger. It also exaggerates the relative size of Europe and gives it pride of place in the middle of the map. As size is all too readily equated with importance, the Mercator projection has often been accused of bias, promoting a Euro-centric world view, colonialism and even racism (Johnson, 2017). Like the Mercator projection, linear frequency scales distort area by over-representing higher frequencies at the expense of lower ones and this distortion inevitably biases our perception of the relative contribution of the different frequency components. Alternatives to the Mercator projection, such as the Peter's Projection, correct the area bias but at the cost of distorting shape. The fundamental problem, of course, is that both the Mercator and Peters projections and TFPs are representations of reality and not reality itself; all models are compromises.

So, how often are linear frequency scales used in TFPs? To answer this question, I conducted a cursory search of original articles published in “*Frontiers of Human Neuroscience*” using search terms “Time-frequency” or “wavelets” and identified 44 articles that included a TFP and, of these, nearly all (41 out of 44 or 93%) used linear frequency scales although many covered only a narrow frequency range where the bias between the highest and lowest frequencies displayed would have been relatively modest. There is no reason to think that “*Frontiers in Human Neuroscience”* is unusual in this respect, nor is the use of linear frequency scales restricted to journal articles. Indeed, linear frequency scales appear to be the norm at scientific conferences and are the default, and sometimes, the only option, in many software packages. In short, linear frequency scales dominate the TFP scene.

Having identified the bias, the solution is straightforward. As EEG/MEG power is proportional to $\frac{1}{f}$, the total power between any two frequency points, for *f* ≠ 0, can be derived from the integral, $\int \frac{1}{f}\;df=\ln|f|+c$. It follows that we can achieve a proportional relationship between frequency and power by adopting a logarithmic scale. Unfortunately, logarithmic scales are not intuitive to most users. A *log*_10_ scale, for example, would cover the entire EEG frequency range in 3 steps (0, 1 and 2 for 1, 10, and 100 Hz) and 2.5, 5, 10, 20, and 40 Hz would be represented by 0.4, 0.7, 1.0, 1.3, and 1.6, respectively. However, octave scales, familiar from music, in which the relationships between frequencies are represented as the base-2 logarithm of the ratio of their frequencies, $log_{2}\left(\frac{f_{2}}{f_{1}}\right),\;$provide a natural fit to the EEG and powers of 2 (1, 2, 4, 8, 16, 32, 64, etc.) involve sequential doublings that are intuitive and easy to understand. Furthermore, it has often been noted that the EEG frequency bands naturally follow a logarithmic scale, with a base value of about 2 although there is a dispute as to whether the base-value is Euler's number, *e* [2.7182 (Penttonen and Buzsáki, 2003)], the Golden Ratio, φ, [1.6180 (Roopun et al., 2008; Pletzer et al., 2010)] or 2 itself (Klimesch, 2013).

Figure 1 illustrates the effect of linear and octave scales on EEG/MEG data. Figure 1A shows a random sample of 2000 data points selected from a $\frac{1}{f}$ power noise distribution (Little et al., 2007) to represent 4 s of EEG/MEG recorded with a sampling rate of 500 Hz and scaled to RMS amplitude of 30 μV and Figure 1B shows the power distribution of the data. Figure 1C shows a Morlet wavelet time-frequency decomposition of the signal with a linear scale. Note the characteristic series of narrow vertical stripes in the gamma range that represent broad-spectrum low amplitude oscillations and that the highest power regions occupy a very narrow band at the bottom of the panel. Figure 1D shows the same data on an octave scale and the advantages of this format are immediately apparent: the high power changes at low frequency are much more easily discernible and, although the narrow vertical stripes in gamma are foreshortened, the high frequency changes remain clearly visible. It is not that “octave” scales are “right” and linear scales “wrong,” they are both representations of reality, but octave scales produce a more equitable distribution between signal power and frequency range than linear scales and thereby reduce the bias toward high frequencies.

**Figure 1:**
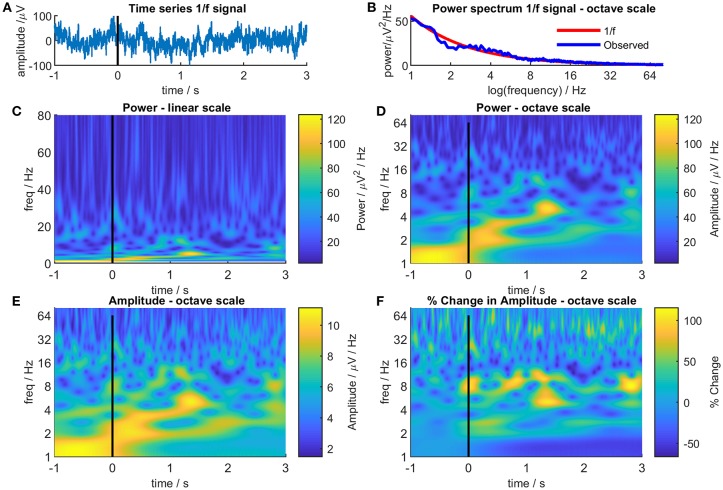
Different representations of 4s of randomly generated 1/f noise. Panel (**A**) shows the time-series of the noise. For illustrative purposes, the time series was treated as a single epoch of EEG/MEG recorded during an even-related paradigm where the time from −1 to 0s was considered as a baseline period and the time from 0 to 3s as the post-event period. Panel (**B**) shows the Fourier power spectrum of the noise (blue line) together with the theoretical 1/f power spectrum (red line). Panel (**C**) shows a Morlet wavelet time-frequency decomposition of the 1/f noise with a conventional linear frequency scale. Panel (**D**) is the same as Panel (**C**) but with an octave frequency scale. Panel (**E**) is the same a Panel (**D**) but showing signal amplitude instead of signal power. Panel (**F**) shows the signal amplitude at each time and frequency as a percentage of the mean signal amplitude at that frequency in the baseline period (−1 to 0s).

There are, however, two objections to the use of octave scales that should be considered. First, a researcher might claim that they are only interested in a narrow range of frequencies or a specific frequency band, not the broad-spectrum from 1 to 80 Hz shown in Figure 1 and that, in such cases, the difference between linear and octave scales is relatively unimportant. There is some truth to this and many of the TFPs previously published in “Frontiers in Human Neuroscience” have used a restricted frequency range, presumably for this reason but some degree of bias will remain. In addition, recent theoretical developments suggest that event-related changes in the EEG/MEG involve co-ordinated synchronization across a broad range of frequencies (Burgess, 2012) and this, together with the increased awareness of the importance of cross-frequency coupling (Canolty and Knight, 2010), suggest the EEG/MEG cannot be properly understood by focusing on a narrow band of frequencies alone. From this perspective, electing to present only a narrow range of frequencies could look like “cherry picking” and becomes difficult to justify.

The second objection, related to the first, is that if a broad-spectrum frequency range is used, the power differential between high and low frequencies means that changes in the higher frequency ranges are obscured regardless of whether a linear or an octave scale is used. Again, there is some truth to this but there are also simple solutions. The color scales in TFPs most commonly represent EEG/MEG power which is positively skewed (lognormal distribution) so the plots tend to be dominated by the colors representing low power with only a few areas showing high power colors. Amplitude is less skewed than power (amplitude = √power), so using color to represent amplitude rather than power [ensures a greater color variation across the TFP and reveals more detail (Figure 1E)].

Using amplitude goes some way to attenuating the differential between high and low frequencies but the problem can be completely overcome by normalizing the color scale for each frequency. As EEG/MEG follows a $\frac{1}{f}$ distribution, weighting each time-frequency point in proportion to its frequency might seem to be a suitable normalizing factor. Unfortunately, this fails to take into account the poorer signal to noise ratio seen at higher frequencies with the result that although the $\frac{1}{f}$ distribution is corrected, noise is disproportionately amplified at higher frequencies. A more conventional approach is shown in Figure 1F which displays the same data as before but this time each pixel represents the percentage change in amplitude based on the mean amplitude in a defined baseline period (in this case, from −1 s to 0 s) at that frequency. Using this simple transformation, a myriad of small, high-frequency changes emerge that were not seen in the previous plots.

The purpose of this opinion piece has been to point out an important bias in the conventional visual representation of time-frequency decompositions and to propose a few simple changes to enhance the effectiveness of TFPs as aids in the interpretation of EEG/MEG data. These changes are to use (i) broad-spectrum frequency ranges, (iii) amplitude instead of power, (ii) frequency-specific normalization of amplitude and, most importantly, (iii) octave frequency scales. TFPs are, after all, simply models of our data and, as George Box's aphorism has it, “*All models are wrong but some are useful*” (Box, 1976) and we should do what we can to enhance their usefulness.

## Author Contributions

The author confirms being the sole contributor of this work and has approved it for publication.

### Conflict of Interest Statement

The author declares that the research was conducted in the absence of any commercial or financial relationships that could be construed as a potential conflict of interest.
